# Genetic Structure, Selective Signatures, and Single Nucleotide Polymorphism Fingerprints of Blue Tilapia (*Oreochromis aureus*), Nile Tilapia *Oreochromis niloticus*), and Red Tilapia (*Oreochromis* spp.), as Determined by Whole-Genome Resequencing

**DOI:** 10.3390/ijms26104910

**Published:** 2025-05-20

**Authors:** Jixiang Hua, Yifan Tao, Siqi Lu, Qingchun Wang, Hui Sun, Yalun Dong, Jun Qiang

**Affiliations:** 1 Wuxi Fisheries College, Nanjing Agricultural University, Wuxi 214081, China; 2 Key Laboratory of Freshwater Fisheries and Germplasm Resources Utilization, Ministry of Agriculture and Rural Affairs, Freshwater Fisheries Research Center, Chinese Academy of Fishery Sciences, Wuxi 214081, China

**Keywords:** tilapia, whole-genome resequencing, genetic diversity and structure, selective features, fingerprinting

## Abstract

Tilapia (*Oreochromis* spp.) is a globally important farmed fish. Analyses of genetic variation across different types of tilapia are essential for the development of superior breeding populations. We investigated the genetic structures of breeding populations of blue tilapia (*Oreochromis aureus*) (OA), Nile tilapia (*Oreochromis niloticus*) (ON), and red tilapia (*Oreochromis* spp.) (OS) by whole-genome resequencing. The results showed that the OS population had maintained high genetic diversity but significant genetic differentiation from the OA population. Principal component analysis, phylogenetic analysis, and genetic clustering analysis revealed a clear pattern of genetic differentiation among the three populations. The genetic structure of the ON population differed from that of the OA population but was similar to that of the OS population. Population kinship analysis revealed a close relationship between the ON and OS populations. Selective scanning analyses of three comparison groups (OA vs. ON, OA vs. OS, and ON vs. OS) revealed population-selected regions related to metabolism, endocrine, and immune systems, harboring key genes (*qrsl1*, *pde4d*, *hras*, *ikbkb*, *prkag1*, *prkaa2*, *prkacb*, *irs2*, and *eif4e2*). These key genes were related to growth, reproduction, and disease resistance, indicating that breeding programs have selected for these traits. Due to the lack of stable morphological characteristics of juvenile fish and the changes in external environmental conditions that lead to changes in individual morphological characteristics, SNP fingerprints were successfully constructed for the identification of the three populations based on the differences in SNPs. Based on the five core SNP markers, two combinations of SNP markers were developed to accurately identify the three populations of tilapia at the genomic level. These results provide new information about tilapia genetic resources and reference data for identification and breeding purposes.

## 1. Introduction

Tilapia (*Oreochromis* spp.) belong to the Cichlidae family in the Perciformes order. These fish originated in Africa but are now widely distributed in tropical and subtropical regions. They are popular species for aquaculture worldwide because of their fast growth, strong reproductive capacity and adaptability, and use as a nutrient-rich food source [1]. China’s total production of farmed tilapia has increased every year, reaching 1.817 million tons in 2023 and making it the top producer globally. Since the 1960s, dozens of tilapia species have been introduced into China, including Mozambique tilapia (*Oreochromis mossambicus*), red tilapia (*Oreochromis* spp.), Nile tilapia (*Oreochromis niloticus*), and blue tilapia (*Oreochromis aureus*). Through continuous artificial selection and crossbreeding, several new tilapia varieties and strains with fast growth, strong disease resistance, and high survival rates have been obtained. For example, *O. niloticus* ♀ × *O. aureus* ♂ is a hybrid that shows strong disease resistance [2]. However, the early sexual maturity and short culture cycle of tilapia have resulted in interspecific hybridization and genetic mixing, which has had a detrimental effect on the quality of germplasm resources. This hinders the sustained and healthy development of the tilapia aquaculture industry [3,4]. Therefore, it is important to analyze the genetic diversity among various breeding populations and to establish an effective and accurate method for germplasm identification. These advances will allow for the conservation and utilization of tilapia germplasm resources and promote the development of the aquaculture industry.

High-quality germplasm resources with wide genetic diversity are required to breed new tilapia germplasm with superior traits, such as high yield, strong adaptability, and resilience. Genetic diversity can be defined as the sum of genetic variations among individuals of different species or populations, and it is a key factor affecting the adaptability of species or populations to environmental changes [5,6]. Molecular markers based on differences in genomic nucleotide sequences have become an important tool for analyzing genetic diversity. Molecular marker techniques such as amplified fragment length polymorphism (AFLP) markers [7], mitochondrial DNA markers [8], and simple sequence repeat (SSR) markers [3] have been used to assess the genetic diversity and genetic structure of wild and farmed populations of tilapia. Studies using these markers have revealed intraspecific mutations and gene penetration in natural populations of tilapia. The use of first- and second-generation molecular markers in analyses of genetic diversity is effective for detecting genetic variation among species, but there are limitations such as poor reproducibility, high costs, and low throughput. Single nucleotide polymorphism (SNP) markers are third-generation molecular markers with several advantages, including a large number of loci, high density within the genome, and genetic stability. In addition, it is easy to automate SNP-based analyses. For these reasons, SNPs have been widely used for marker-assisted selection and to construct genetic linkage maps, identify germplasm resources, and detect genetic diversity in a wide range of aquatic animals [5,9,10].

With the rapid development of next-generation sequencing (NGS) technology and the publication of reference genome sequences for more species, mining for high-density and high-quality SNP markers at the whole genome level has become a mainstream method for analyzing the genetic diversity of aquatic animals [11]. In particular, whole-genome resequencing (WGR) technology is often used to detect differences in the genetic diversity of aquatic animals from different selective populations or among populations from different geographic areas. Previous studies have used WGR technology to analyze the genetic diversity of largemouth bass (*Micropterus salmoides*) [12], mandarin fish (*Siniperca chuatsi*) [13], and large yellow croaker (*Larimichthys crocea*) [14]. Xia et al. (2015) [15] used WGR sequencing to analyze the genetic diversity of genetically improved farmed tilapia (GIFT tilapia), Nile tilapia, red tilapia, and wild Mozambique tilapia in China. They found that artificially selected GIFT tilapia had the lowest genetic diversity and wild species had the highest [15]. In addition, WGR has been used in genetic studies on foreign native tilapia populations and different strains of Nile tilapia [16,17,18].

Studies using WGR technology can provide insights into the evolutionary mechanism of population selection, because this technology detects features at the genome level that have been selected for during the process of adaptation to the natural environment or artificial selection. For example, selective scanning analyses revealed that genes associated with growth, development, and immunity have been selected for domestically bred largemouth bass [19]. Similarly, analyses of the genetic changes brought about by artificial selection of mono-female grass carp (*Ctenopharyngodon idella*) and wild grass carp revealed that candidate genes related to gonadal development and growth have been selected for [20]. Nile tilapia, the tilapia species most recently introduced into China, was brought in by our research group from St. Louis, Senegal, in 2023. Studies on the mechanism of selective adaptation during the process of stock introduction and domestication can yield information about stock-specific genetic characteristics and provide a reference for a scientific and rational breeding strategy.

In this study, we explored the genetic diversity and population structure of the most recent selected populations of blue tilapia, red tilapia, and Nile tilapia (*T. aureus*, *Tilapia* spp., and *T. niloticus*), which were introduced into China in 2004, 2010, and 2023, respectively. Whole-genome resequencing was conducted, and a selective clearance analysis was used to identify the genetic regions and candidate genes under selection in the three populations. Finally, SNP marker fingerprints were constructed on the basis of genetic loci showing SNP variations among populations. The overall aim of this research was to provide a scientific basis for the improvement of tilapia germplasm and for accurate germplasm identification.

## 2. Results

### 2.1. Whole-Genome Resequencing Data Statistics

Sequencing libraries were constructed for 45 individuals from three different tilapia populations for whole-genome resequencing. After quality control, 106,575,864, 135,828,530, and 116,694,036 high-quality reads were obtained for the OA, ON, and OS populations, respectively. The ON population had the highest detection rate of high-quality reads, but the lowest rate of read mapping to the reference genome (94.20%). The mapping rate of reads to the reference genome was higher than 97% for the OA and OS populations. Across the three populations, the ranges of average GC content, Q20, and Q30, were 41.78–43.08%, 97.12–98.89%, and 94.84–97.02%, respectively. The average sequencing depth for the three populations was 4.19× (Table S3). Overall, these results indicated that the sequencing data were of sufficient quality for SNP analysis.

### 2.2. Whole-Genome SNP Statistics and Genetic Diversity Analysis

**Figure 1:**
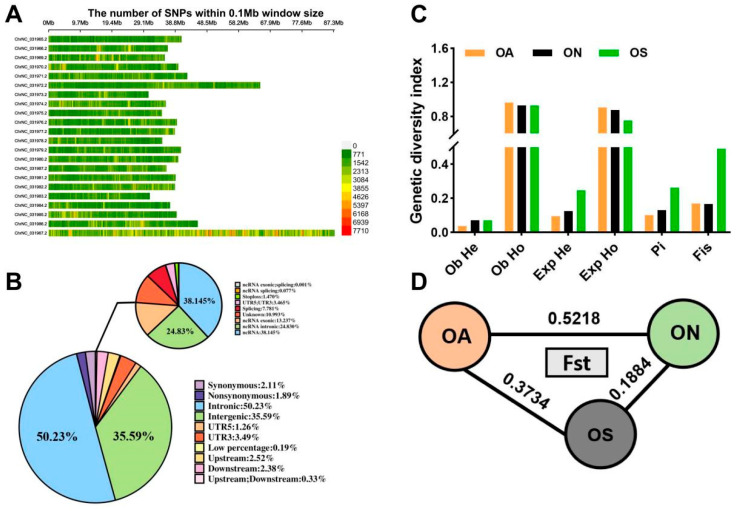
Population-wide SNP screening and statistics and analysis with genetic diversity. (**A**) Distribution of SNPs on chromosomes, the change in color from green to red indicates an increase in SNP density at 0.1 Mb, with stronger colors representing higher SNP densities. (**B**) Statistics of SNPs annotated to different regions of the genome. (**C**) Population genetic diversity index statistics. (**D**) Population fixation index of the three species.

After filtering using GANK software, 66,176,171, 122,139,799, and 93,312,060 SNPs were detected in the OA, ON, and OS populations, respectively. The SNP detection rate was significantly higher in the ON population than in the OA and OS populations. The ON population had the lowest transition to transversion ratio (Ts/Tv of 1.72) (Table S4). The SNPs were unevenly distributed over 22 chromosomes. On the basis of the number of SNPs in a window of 0.1 Mb, the highest SNP coverage was in the ChrNC_031967.2 genomic sequence (Figure 1A). According to annotation information, 50.26% of the SNPs were located in introns, 35.61% were in intergenic regions, and 4.05% were in exons (Figure 1B).

Further analyses of differences in genetic diversity among the three populations revealed that the OS population had the highest mean observed heterozygosity (0.0711) and mean expected heterozygosity (0.2469), and the OA population had the lowest values for these indexes (0.0370 and 0.0954, respectively). The OA population had the highest mean observed homozygosity (0.9630) and mean expected homozygosity (0.9046), and the OS population had the lowest values for these indexes. *Pi* is an important parameter for measuring genetic diversity within a population, was highest in the OS population (0.2626) and lowest in the OA population (0.1017). Finally, the *F_IS_* was much higher in the OS population than in the OA and ON populations (Figure 1C). Overall, the OS population maintained high genetic diversity. The *F_ST_* value is indicative of the degree of genetic differentiation between populations; there was a low degree of genetic differentiation between the OS and ON populations (*F_ST_* = 0.1884) and a high degree of genetic differentiation between the OA and ON populations (*F_ST_* = 0.5218) (Figure 1D).

### 2.3. Population Genetic Structure Analysis

**Figure 2:**
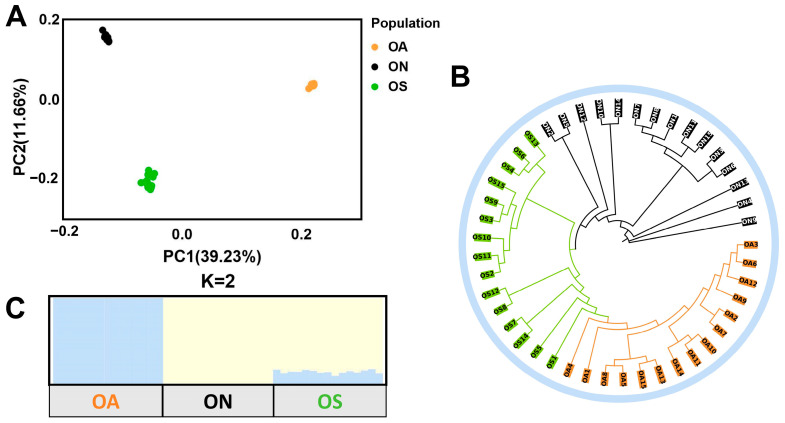
Genetic structure of three tilapia populations. (**A**) Population principal component analysis. (**B**) Population phylogenetic tree. (**C**) Genetic structure of three populations with K = 2. Each individual in the population is represented by a column, with different colors representing the proportion of shared ancestry.

The PCA results showed that individuals from each of the three populations were tightly clustered, indicating no significant divergence differences among individuals, with PCA1 explaining 39.23% of the variance and PCA2 explaining 11.66% of the variance. These reflected the pattern of divergence between the wild species and the selections, where the ON population showed a clear segregation from the OA and OS populations, and the OS population was in the center, which was in line with its hybridization origins pattern (Figure 2A). This was also confirmed by the results of the phylogenetic tree, which showed that all the individuals were mainly divided into two branches, the ON population formed a branch of its own, and the OS and OA populations shared a branch, but formed their own clustering patterns, further indicating that there was no gene infiltration in the ON wild population, whereas the branch of the OS population was closely connected to the ON population, suggesting that the gene infiltration of the OS population was closer to that of the ON population, which was in line with its hybrid origin, and that the same branch with the OA population might have originated from partial gene infiltration (Figure 2B). As shown in the plot of K values of 1–10 against CV error values, K = 2 had the lowest error (CV = 0.63), so K = 2 was used as the optimal number of groups for clustering (Figure S1A). When K = 2, both the OA and ON groups formed independent groups, and the OS group was mixed with the ON group, indicating a common ancestral origin, responding to its genomic characteristics as a hybrid (Figure 2C and Figure S1B). Thus, the results of the admixture analysis were consistent with the results of the PCA and the phylogenetic analysis and proved that the OS and ON populations were more similar in their population genetic structures.

### 2.4. Population Kinship, Gene Flow, and Linkage Disequilibrium Analysis

**Figure 3:**
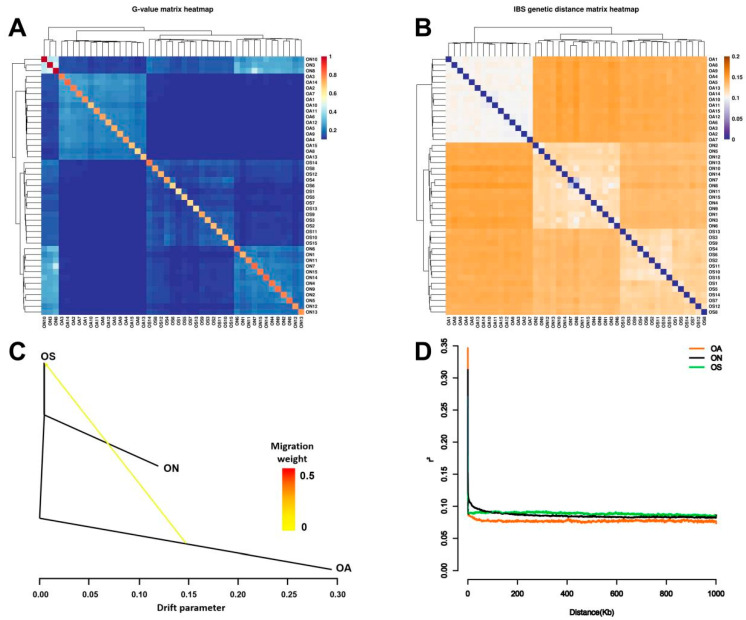
Population kinship with gene flow and linkage disequilibrium analysis. (**A**) G-value matrix indicating kinship (relatedness). (**B**) IBS genetic distance matrix indicating degree of inter-individual variation. (**C**) Gene flow analysis expressed as a maximum likelihood tree—yellow arrow indicates gene migration events and direction of arrow indicates direction of gene drift. (**D**) Rate of LD decay for OA, ON, and OS populations.

Genome-wide relationship and IBS matrices were constructed to explore the kinship of the three populations. In the G-value genetic distance matrix, which represents the degree of kinship (relatedness), the range of values between OA/ON, OA/OS, and ON/OS individuals was 0.141–0.152, 0.138–0.146, and 0.130–0.142, respectively (Figure 3A). In the IBS genetic distance matrix, which represents the degree of inter-individual variation, the range of values between OA/ON, OA/OS, and ON/OS individuals was 0.074–0.150, 0.065–0.117, and 0.086–0.203 (Figure 3B). Thus, in general, there were close relationships among the three populations, but especially between the ON and OS populations.

Genome-wide allele frequencies were used to infer splitting and mixing events in the three populations. In the maximum likelihood tree, the OS and ON populations were clustered into a single unit, and the OS population formed a distinct unit. However, genetic drift from the OA population to the OS population was evident (Figure 3C), indicating that genetic material from the OA population introduced genetic variation into the OS population. In the LD analysis, the LD coefficients (r^2^) of the ON and OS populations exhibited minimal variation over a distance of 1000 kb. The lowest r^2^ of 0.075 was observed for the OA population. The LD decay rate was fastest for the OA population and slowest for the OS population (Figure 3D).

### 2.5. Identification of Selected Regions in Genomes of the Three Populations and Candidate Gene Analysis

The population-selected regions were identified in pairwise comparisons of the groups (OA/ON, OA/OS, and ON/OS), with the top 5% of the absolute values of the θπ ratio and *F_ST_* values serving as thresholds. We detected 262 selected regions and 204 annotated candidate genes in the OA/ON comparison group; 393 selected regions and 247 annotated candidate genes in the OA/OS comparison group; and 3348 selected regions and 1452 annotated candidate genes in the OS/ON comparison group (Figure S2).

**Figure 4:**
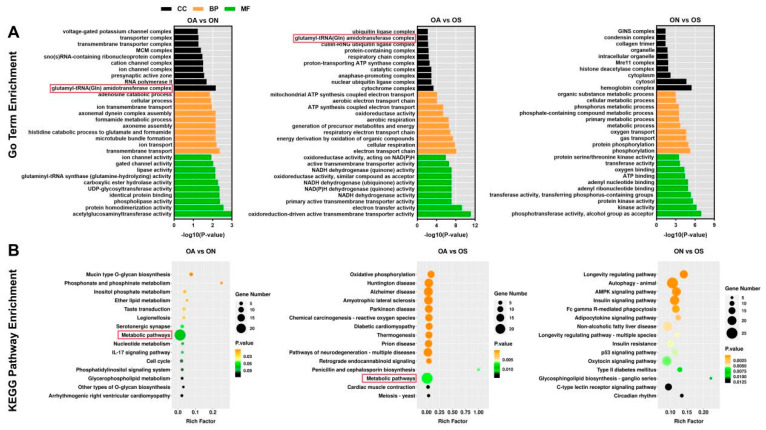
GO and KEGG enrichment analysis of candidate genes in selected regions in the three populations. (**A**) GO enrichment analysis of candidate genes detected in OA vs. ON, OA vs. OS, and ON vs. OS comparisons. Top 10 biological process (BP), top 10 cellular component (CC), and top 10 molecular function (MF) terms are shown. Red box highlights glutamyl-tRNA(Gln) amidotransferase complex GO term, which was enriched with candidate genes detected in OA vs. ON and OA vs. OS comparison groups. (**B**) KEGG pathway enrichment analysis based on candidate genes detected in OA vs. ON, OA vs. OS, and ON vs. OS comparisons. Top 15 enriched pathways in each of the three comparison groups are shown. Red box indicates ‘metabolic pathways’, which were enriched with candidate genes detected in OA vs. ON and OA vs. OS comparisons.

The candidate genes in the selected regions of the genome in the three comparison groups were subjected to GO term and KEGG pathway enrichment analyses. The top 10 enriched GO terms (*p* < 0.05) in the biological process (BP), cellular component (CC), and molecular function (MF) categories are shown in Figure 4A. In the CC category, glutamyl-tRNA(Gln) amidotransferase complex, cytochrome complex, and hemoglobin complex were among the top GO terms significantly enriched with candidate genes identified in the OA/ON, OA/OS, and ON/OS comparisons. The glutamyl-tRNA(Gln) amidotransferase complex GO term in the CC category was significantly enriched with candidate genes identified in the OA/ON and OA/OS comparisons. In the BP category, transmembrane transport, electron transport chain, and phosphorylation were the GO terms significantly enriched with candidate genes detected in the three comparison groups. In the MF category, acetylglucosaminyltransferase activity, oxidoreduction-driven active transmembrane transporter activity, and phosphotransferase activity were significantly enriched GO terms.

Next, KEGG analyses were conducted to identify pathways enriched with candidate genes detected in the pairwise comparisons. The top 15 enriched pathways are shown in Figure 4B. These ‘metabolic pathways’ were co-enriched with genes detected in the OA/ON and OA/OS comparisons, indicating that metabolism-related pathways play an important role in the genetic improvement of the OA population. Numerous pathways related to aging, the endocrine system, and the immune system were significantly enriched with candidate genes detected in the ON/OS comparison, such as the longevity regulating pathway, the insulin signaling pathway, and the C-type lectin receptor signaling pathway.

**Figure 5:**
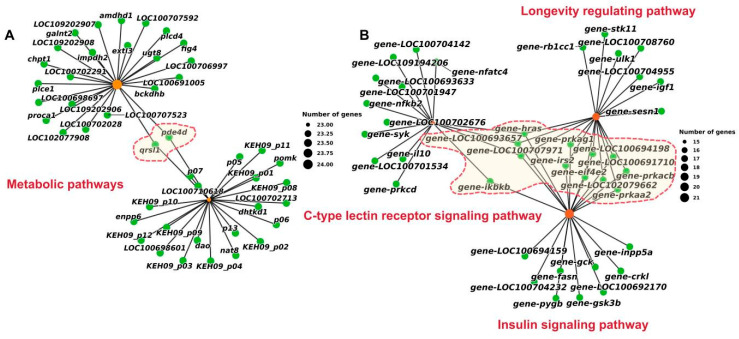
Network diagrams of pathways enriched with candidate genes. (**A**) Network diagram of genes in metabolic pathways detected in OA vs. ON and OA vs. OS comparisons; red dashed line indicates common genes. (**B**) Network diagram of genes involved in aging-, endocrine system- and immune system-related pathways detected in ON vs. OS comparison; red dashed line indicates common genes.

Further targeting the KEGG pathway mentioned above, a gene–pathway enrichment network was mapped to identify genes shared among different pathways. As shown in Figure 5A, *qrsl1* and *pde4d* were common genes in ‘metabolic pathways’ in both comparison groups. In the ON/OS comparison group, *hras* was shared by three pathways, and *ikbkb*, *prkag1*, *prkaa2*, *prkacb*, *irs2*, and *eif4e2* were also identified as shared candidate genes (Figure 5B). These eight genes were identified as core candidate genes in the selected regions of the three populations.

### 2.6. SNP Fingerprint Construction and Validation

**Figure 6:**
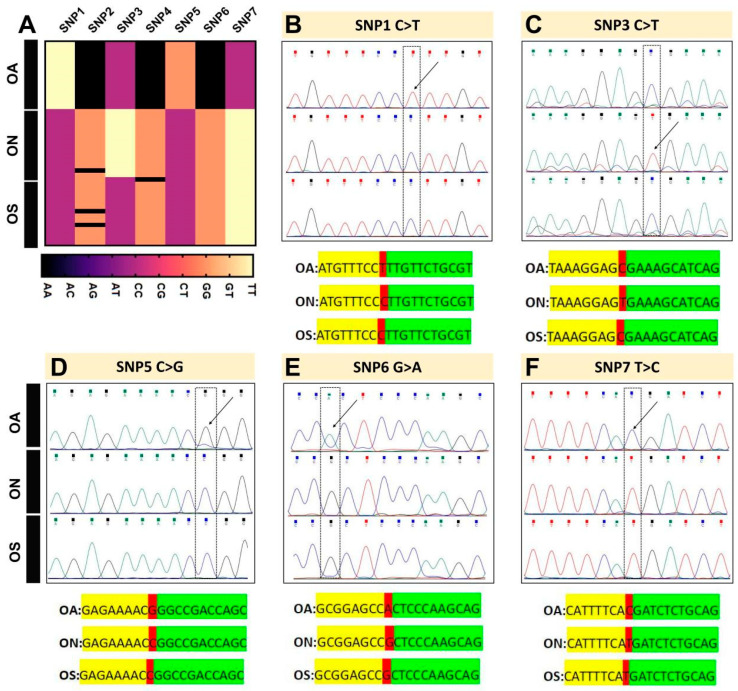
Construction and validation of SNP fingerprints for the three populations. (**A**) DNA fingerprints of three populations constructed based on seven core SNPs, each row represents one individual, each column represents one core SNP, and the genotypes of the individuals are differentiated by different colors: TT (yellow), AA (black), GG (orange), and CC (purple). (**B**–**F**) SNP typing by Sanger sequencing and comparison of amplified sequences. Peak maps show positions of mutations (shown in black dashed boxes and highlighted with black arrows); sequence mutation site is highlighted in red.

From the filtered high-quality SNPs, seven core SNP loci were selected according to the screening criteria (Table S5). On the basis of the genotyping results of the OA, ON, and OS populations at the core SNP loci, SNP fingerprints were constructed for 45 individuals from the three populations. As shown in Figure 6A, each row represents an individual (45 rows), each column represents a core SNP (7 columns), and the genotype of each individual is distinguished by different colors. Four pure genotypes were evident in the fingerprinting profile: TT (yellow), AA (black), GG (orange), and CC (purple). For example, using the SNP1 marker to genotype the three populations, the genotype of the OA population was TT, and that of the OS and ON populations was CC; thus, the SNP1 marker could initially distinguish the OA population. Similarly, the genotypes at the markers SNP3, SNP5, SNP6, and SNP7 distinguished one of the three populations from the other two. However, for markers SNP2 and SNP4, some individuals in both the OS and ON populations exhibited the AA genotype (black) instead of the GG genotype (orange), and thus, they did not allow for initial distinction among the three populations.

To verify the accuracy of the fingerprint profiles, forty additional samples were collected from each of the three populations. Specific amplification primers were designed for the seven core SNP markers, and Sanger sequencing was conducted. The sequences were used for SNP typing and for comparisons among individuals. An example is shown in Figure 6B, where the Sanger sequencing results revealed a C to T base mutation at marker SNP1. At the SNP1 locus, the OA population was homozygous TT, corresponding to T single peaks, whereas the ON and ON populations were homozygous CC, corresponding to single peaks. The peaks were clear and there were no peaks indicative of heterozygosity. Sequence comparison showed that the marker SNP1 had a C/T polymorphic locus, consistent with the fingerprinting results, and thus the SNP at this locus could be used as a marker for population identification. The markers SNP3, SNP5, SNP6, and SNP7 had C > T, C > G, G > A, and T > C base mutations, respectively (Figure 6C–F), and the validation results were consistent with the fingerprinting patterns. However, the sequencing results of SNP2 and SNP4 had G > A mutations in the OS and ON populations, and thus, these SNPs could not be used as markers to identify populations.

### 2.7. Combinations of SNP Markers for Population Identification

**Figure 7:**
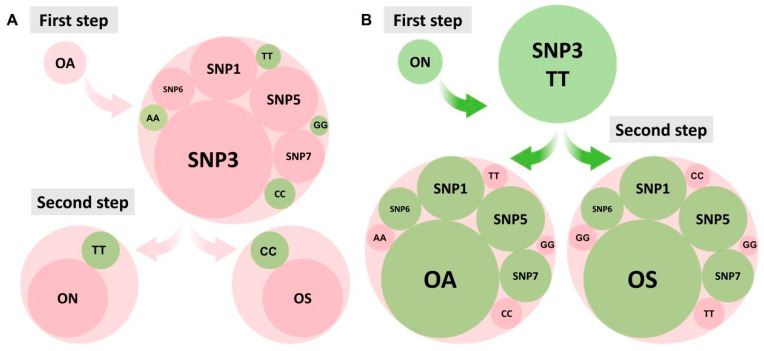
Step-by-step bead-wrap diagram for identification of the three tilapia populations. (**A**) First marker combination: step 1, four SNP markers are used to identify blue tilapia; step 2, SNP3 distinguishes between Nile tilapia and red tilapia. Pink labels indicate SNP markers and green labels indicate genotypes. (**B**) Second marker combination: step 1, SNP3 identifies Nile tilapia; step 2, four SNP markers distinguish between blue tilapia and red tilapia. Green labels indicate SNP markers and pink labels indicate genotypes.

We determined the optimal combinations of markers SNP1, SNP3, SNP5, SNP6, and SNP7 to identify the different populations. Two combinations of markers successfully identified the three populations. For both marker combinations, a two-step process was used to distinguish the populations (Figure 7A). For the first combination of markers, the first step identified blue tilapia with SNP1 (TT genotype), SNP5 (GG genotype), SNP6 (AA genotype), and SNP7 (CC genotype). In the second step, the SNP3 marker distinguished Nile tilapia (TT genotype at SNP3) from red tilapia (CC genotype at SNP3). For the second marker combination, SNP3 distinguished Nile tilapia SNP3 (TT genotype) from the other two populations, and then SNP1, SNP5, SNP6, or SNP7 distinguished between blue tilapia and red tilapia. For example, using SNP1 as the marker, an individual with the TT genotype was identified as blue tilapia, and an individual with the CC genotype was identified as red tilapia (Figure 7B). Both marker combinations accurately identified blue tilapia, Nile tilapia, and red tilapia.

## 3. Discussion

### 3.1. Genetic Diversity and Genetic Structure

Tilapia is a globally distributed freshwater aquaculture species with more than 600 documented species worldwide. China has emerged as a leader in the field of tilapia aquaculture, and numerous varieties have been developed through new introductions and selective breeding. Understanding the variations in genetic diversity among species and populations is a prerequisite for developing successful breeding strategies. Tilapia are typically sexually dimorphic, with males growing faster and having a higher economic value compared with females [21]. Blue tilapia is a pivotal parent species in the production of all-male tilapia through crossbreeding with Nile tilapia, where the ratio of male offspring can exceed 90% [22]. Nile tilapia, with superior culture performance and strong environmental tolerance, is the main cultured tilapia species in China and is an important cornerstone for the improvement of germplasm resources [23,24]. Red tilapia is obtained by crossing mutant red Mozambique tilapia with other tilapia species, including Nile tilapia. Red tilapia has excellent breeding potential because of its bright body color, appealing flavor, and high salinity tolerance [25,26,27]. Therefore, in this study, we conducted WGR and analyzed the sequences to detect genetic differences at the genome-wide level among blue tilapia, red tilapia, and the newly introduced Nile tilapia.

Heterozygosity is an important measure of genetic polymorphism [28]. In this study, the mean observed and expected heterozygosity values were highest in the OS population and lowest in the OA population. The OA population used in this study was selected over 20 consecutive generations. Artificial directional selection causes fluctuations in the level of genetic diversity within the population. The lower level of heterozygosity in the OA population suggests that the level of genetic variation within the population has decreased, i.e., consecutive selection has reduced the genetic diversity of this population. Consistent with this result, other studies have detected reductions in heterozygosity during the artificial selection of largemouth bass, grass carp, and blunt snout bream (*Megalobrama amblycephala*) [29,30,31]. The OS population has also been under continuous selection but has maintained a high degree of heterozygosity, which may be related to its hybrid advantage. Crossbreeding advantage is one of the most important ways of obtaining varietal improvement in plant and animal species, and hybrids can transfer beneficial parental traits to increase the degree of genetic variation in the offspring [32]. We detected a higher degree of heterozygosity in the OS population than in the ON population, which is a side effect of the higher degree of genetic variation in hybrids. The observed heterozygosity was lower than the expected heterozygosity in all three populations, suggesting that all three populations are heterozygous to some degree. This may be related to null alleles, unbalanced sex ratios, inbreeding, and excessive artificial selection [33]. The OA population had the highest mean values of observed and expected homozygosity, indicating that it has consistently maintained homozygosity, and thus is more favorable for the continuous improvement of target traits such as growth.

A previous study found that the high degree of heterozygosity of the Taiwanese red tilapia population was associated with a preference for individuals with a more orange-red body color during breeding selection [8]. Compared with individuals in the OA and ON populations, those in the OS population had a more pinkish or whitish-pink body color. The degradation of body color might be one of the reasons for the reduced homozygosity of the OS population. Nucleotide diversity (*Pi*) represents the level of variation within the genome, and the inbreeding coefficient (*F_IS_*) indicates the degree of inbreeding within the population [28]. The *F_IS_* of the OS population was much higher than that of the ON and OS populations, and the *Pi* of the OS population was approximately double that of the other populations. The high *Pi* value indicates higher genetic diversity in the population, but the high *F_IS_* value indicates inbreeding within the OS population. OS is a superior species obtained by crossing Mozambique tilapia with ON and other species; the hybridization advantage increases its level of genetic variability. The family line selection method was used for OS population selection. The use of this method meant that the OS population consisted of far fewer offspring compared with the ON and OA populations. Therefore, long-term artificial selection may have led to an increase in the inbreeding coefficient, but ancestral polymorphisms introduced by crossbreeding likely contributed to its high genetic diversity. In the context of genetic enhancement of OS in the future, it is important to use appropriate breeding methods to mitigate the likelihood of inbreeding decline [34].

Geographical distance, the environment, and artificial selection all contribute to the genetic differentiation of populations [13,35]. The *F_ST_* value is a crucial metric for evaluating the degree of genetic differentiation within a population [5]. In this study, we detected significant genetic differentiation between the OA population and the OS and ON populations (*F_ST_* > 0.25), and less genetic differentiation between the OS and ON populations. The ON population was introduced from Senegal in 2023, and the geographical isolation between the two populations, as well as interspecific differences, has contributed to its genetic differentiation. It is interesting to note that the OS and ON populations show a close relationship and appear to share a common ancestor. In the PCA and phylogenetic tree, the three populations were clearly separated, but the optimal number of subpopulations (*K*) was 2, which divided the three populations into two distinct subpopulations. This finding provides further evidence that the OS and ON populations originated from the same ancestor and have close group kinship.

The range of genetic distances and G-value variations showed that the genetic variations among the three populations were small. However, the maximum likelihood tree showed a genetic drift event from the OA population to the OS population, consistent with the population genetic clustering results. Another study detected gene infiltration from blue tilapia to the Abbassa strain of Nile tilapia and suggested that the mixing of a limited number of individuals at the aquaculture facility may have contributed to this effect [24]. Linkage disequilibrium analyses reveal the degree of selective domestication of populations; the LD level is affected by genetic drift between populations, selection, and recombination frequencies [36,37]. In this study, the OA population had the fastest rate of decay and the lowest level of LD, in contrast to the OS population. This suggests that the OS population is more domesticated and has been under stronger selection intensity. Notably, the OS population had the highest level of genetic diversity and the highest level of LD. High LD levels are associated with more inbreeding and a high selection intensity, which may lead to high genetic diversity in the population [38]. The high inbreeding coefficient of the OS population may explain this phenomenon, with similar results reported for largemouth bass [12].

In general, plants and animals have been domesticated over a long period of time, leading to a reduction in the genetic diversity of domesticated species compared with wild species because of bottleneck effects, artificial selection, and selective removal [39,40,41]. In this study, the latest introduced Nile tilapia population exhibited low genetic diversity. The long-term artificial selection in breeding programs may be responsible for its reduced genetic diversity.

### 3.2. Genes Selected in the Three Populations and Their Pathways and Mechanisms

In this study, genetic regions that have been selected in the three populations, and the candidate genes within these regions, were identified on the basis of the combination of θπ and *F_ST_* values. The genes in the selected regions identified in the OA/ON and OA/OS comparisons were subjected to GO term and KEGG pathway enrichment analyses. The GO term glutamyl-tRNA(Gln) amidotransferase complex and ‘metabolic pathways’ were co-enriched with the candidate gene *qrsl1*, which encodes GatA, a component of the glutamyl-tRNA(Gln) amidotransferase complex. This complex is involved in glutamyl-tRNAGln biosynthesis and contributes to synthetase activity through transamidation and mitochondrial translation. The glutamyl-tRNA(Gln) amidotransferase complex is also associated with oxidative phosphorylation in organisms and is closely linked with ATP synthesis. Defects in oxidative phosphorylation contribute to numerous mitochondrial diseases [42]. Sexual differentiation in bony fishes undergoes adaptive changes in response to environmental stresses such as temperature, light, and pollutants [43]. In a study on sex differentiation in Nile tilapia, *qrsl1* was identified as a key target gene regulating oocyte development [44]. Similarly, in Plateau fish (*Triplophysa dalaica*) undergoing selection during environmental adaptation to hypoxia and low temperatures at altitude, *qrsl1* was identified as a key gene associated with the response to hypoxia [45].

The metabolic pathways network included *pde4d*, which encodes a protein in the phosphodiesterase family that plays a role in the degradation of cyclic adenosine monophosphate (cAMP). Through the activity of activation-dependent protein kinase A (PKA), cAMP is used to phosphorylate downstream target proteins, thereby modulating cellular function in the inflammatory response. The antimicrobial peptide, tilapia hepcidin 2-3 (TH2-3), isolated from tilapia, was shown to inhibit pro-inflammatory cytokines through a PDE4D lysogenic mechanism [46]. In rainbow trout (*Oncorhynchus mykiss*), a high expression level of *pde4d* was associated with a reduction in high-fat feed-induced hepatic inflammatory responses mediated through the cAMP pathway [47]. During ovarian development in bony fish and mammals, the cAMP pathway is influenced by gonadotropic regulatory hormones, and it plays an important role in follicular growth and maturation [48]. In mice, specific defects in PDE4D impaired the cAMP signaling pathway, resulting in reduced growth and fertility [49]. In tiger puffer (*Takifugu rubripes*), *pde4d* was identified as an important growth-related circRNA [50]. These results show that metabolic pathways are significantly enriched in the three tilapia populations, and genes associated with traits such as reproduction, disease resistance, and growth have been selected for breeding programs.

Among all the pairwise comparisons, the comparison between the ON and OS populations yielded the largest number of selected regions and genes, which is indicative of significant differences in selective adaptation between these two populations. These two populations show clear differences in aging, endocrine and immune system functions, cell signaling, metabolic capacity, and environmental adaptation. We focused on longevity regulation, insulin signaling, and C-type lectin receptor signaling pathways for candidate gene screening. *hras* was identified as a shared candidate gene across these three pathways, and *ikbkb*, *prkag1*, *prkaa2*, *prkacb*, *irs2*, and *eif4e2* were also identified as shared candidate genes. *hras* belongs to the RAS oncogene family and encodes the H-Ras protein, which mediates several pro-survival and anti-apoptotic signaling pathways, including the PI3K, FOXO, and mTOR pathways [51]. Ras mutations have been identified in assays involving exposure to toxic compounds in fish, and strongly affect cell growth and signaling [52]. The overexpression of *hras* in the Epithelioma papulosum cyprinid cell line led to the early senescence of these cells, but not complete cellular senescence [53]. Although little is known about the function of *hras* in tilapia, we hypothesize that it may significantly influence cell proliferation in Nile tilapia and red tilapia, reducing cellular senescence and thereby extending the lifespan of these fish. *ikbkb* was co-enriched in both the endocrine and immune system pathways. This gene encodes a subunit of the IκB kinase complex and plays a crucial role in activating the NF-κB classical pathway. Upon phosphorylation, activated NF-κB can freely enter the nucleus, where it affects various essential cellular functions, including the immune response, apoptosis, and proliferation [54]. In an experiment where *Gambusia affinis* was exposed to both polystyrene nanoplastics and perfluorooctanoic acid, *ikbkb* expression significantly increased, thereby activating immune- and inflammation-related pathways [55]. *eif4e2* is involved in the negative regulation of protein translation initiation and is frequently used as a marker in studies of Pacific white shrimp (*Penaeus vannamei*) [56,57]. *prkag1*, *prkaa2*, and *prkacb* encode subunits of AMP-activated protease, which plays a crucial role in various energy metabolic processes. This kinase participates in the fatty acid biosynthesis pathway and is involved in the regulation of energy homeostasis. The involvement of *prkag1*, *prkaa2*, and *prkacb* in energy metabolism has been demonstrated in studies on hepatic lipid metabolism in grass carp [58], the liver hypoxia response in greater amberjack (*Seriola dumerili*) [59], and variations in growth rates in *Sinocyclocheilus grahami* (Cypriniformes, Cyprinidae) [60]. Similarly, *irs2* is involved in the regulation of growth- and development-related pituitary hormones, such as insulin and insulin-like growth factor 1. Compared with red tilapia, Nile tilapia shows superior growth performance and higher feed utilization efficiency. On the basis of the genes that have been selected for, the trait of growth performance has been prioritized in the process of genetic breeding improvement.

### 3.3. SNP Fingerprint Construction

There are numerous tilapia species in China, and interspecific hybridization and genetic mixing are inevitable in the process of genetic improvement. Consequently, methods that can accurately identify germplasm resources are urgently needed. In this study, a total of seven core SNP markers were screened and DNA fingerprinting profiles were successfully constructed for the identification of ON, OA, and OS. After the validation of the fingerprint profiles, two combinations of markers for stock identification were determined on the basis of five core SNP markers. Previous molecular markers for identifying tilapia germplasm resources have included RAPD markers [61] and SSR markers [3]. While these methods can identify specific germplasm resources, they also have drawbacks, including complex procedures, high costs, and low throughput. The use of SNP molecular markers addresses these limitations. Currently, SNP marker-based fingerprints are primarily used for the identification of germplasm resources of plants including maize (*Zea mays* L.), cucumber (*Cucumis sativus* L.), and radish (*Raphanus sativus* L.) [62,63,64]. In aquatic animals, Liang et al. developed a set of fingerprints using 15 core SNP markers to accurately identify six species of Chinese soft-shelled turtle (*Pelodiscus sinensis*) from geographically diverse populations [5]. Cui et al. constructed SNP fingerprints of Loach (*Misgurnus anguillicaudatus*) from six different regions to provide technical support for germplasm identification of wild loach [65]. Zhang et al. successfully developed a set of fingerprints based on SNP markers to accurately identify the Pacific oyster (*Crassostrea gigas*) from China, Japan, and Korea, which provides a reliable tool for the accurate identification of oyster germplasm in China [66]. These results progressively proved the importance of SNP fingerprinting in the identification of aquatic animal germplasm resources.

Although the SNP fingerprints constructed from five core SNP markers accurately identified the three populations in this study, there are some limitations. First, the core SNP markers screened here were determined using a sample size of *n* = 15 for each population. The small sample size means that minor variants may have been missed, and it may have led to biases in allele frequency estimation. Thus, further studies using larger validation populations will be useful to confirm the accuracy of these fingerprinting profiles. In addition, the SNP fingerprint profiles developed in this study identified the confirmed populations accurately, but the low sequencing depth may have led to false positives during SNP calling. Therefore, in further studies, increasing the sequencing depth and increasing the number of core SNP markers are two important objectives to improve the accuracy of fingerprinting profiles in identifying target populations. This study is the first to develop SNP fingerprints for identifying ON, OA, and OS. This advancement provides significant support for the future identification of tilapia species and for the genetic enhancement of desirable traits.

## 4. Materials and Methods

### 4.1. Sample Collection

The three tilapia populations used in this study were reared and domesticated by our research group at the Freshwater Fisheries Research Center, Chinese Academy of Fishery Sciences (FFRC) (Wuxi, China). All three tilapia culture populations were cultured in a temperature-controlled indoor recirculating water culture system with the water temperature maintained at ≥28 °C and with continuous oxygenation of the holding water. The dissolved oxygen (DO) concentration in the water was maintained at ≥7.5 mg/L, total ammonia nitrogen at ≤0.3 mg/L, and nitrite at ≤0.1 mg/L. The fish were fed twice daily (8:00 a.m. and 4:30 p.m.) with the same feed, and the culture water was replaced twice per month to ensure clean water. Blue tilapia (OA) was introduced by the FFRC from the Abbassa World Fish Center in Shagia, Egypt, in 2004. Since its domestication in China, it has been under continuous population selection using growth rate and resistance as selection indexes. At the start of the study, it had been continuously selected for 20 generations. Nile tilapia (ON) was introduced by the FFRC from St. Louis, Senegal, in 2023, and it is a wild species that has not been genetically selected. Red tilapia (OS) was introduced from Taiwan, China, by the FFRC in 2010. Initially, 2000 individuals with a pink body surface and without red and black spots were selected from the Taiwanese population as the base population, and body color and body weight were selected as target traits. The family selection technique was used for successive selections up to the 10th generation. In 2024, after determining the sex by observing the urogenital pores in vitro, 15 one-year-old experimental fish (7–8 females) were collected from each of the three populations. These fish had an average size of 530 ± 5.0 g (OA), 750 ± 5.0 g (ON), and 600 ± 5.0 g (OS). Caudal fin samples (0.5 g) were clipped, immediately placed in anhydrous ethanol, and stored at −20 °C until later use.

### 4.2. DNA Extraction, Library Construction, and High-Throughput Sequencing

DNA was extracted from the caudal fin samples using a fully automated nucleic acid extractor coupled with a genomic DNA extraction kit (Novozymes Biotechnology Co., Ltd., Nanjing, China). The concentration and purity of DNA were detected by 1% (*w*/*v*) agarose gel electrophoresis and by using a NanoDrop 2000 spectrophotometer (NanoDrop Technologies, Wilmington, DE, USA). The extracted qualified DNA was subsequently diluted to a concentration of 50–70 ng/μL and then randomly fragmented by ultrasonication. The DNA fragments were then end-repaired according to the library construction standard of the TruSeq kit (Illumina, San Diego, CA, USA). The library templates were enriched by adding sequencing splices at the 5′ end and repairing at the 3′ end, followed by the purification and PCR amplification of the DNA fragments. Fragments of around 450 bp were used to construct libraries. Each library was subjected to 2 × 150 bp double-end sequencing on the Illumina NovaSeq platform by Personal Biotechnology Co., Ltd. (Nanjing, China).

### 4.3. Whole Genome SNP Screening

To obtain high-quality sequences, the raw data were filtered using the fastp tool (https://github.com/OpenGene/fastp, accessed on 6 October 2024) to remove splice sites and low-quality reads. Filtered high-quality reads were compared with the reference genome using the default parameters of the bwa-mem (0.7.12-r1039) program. To improve the accuracy of SNP prediction, Genome Analysis Toolkit (GATK, 4.0.4.0) software was used to detect and confirm SNPs using the following steps: first, the Realigner Target Creator command in the GATK software (version 4.2.1.0) package was used to output a file containing all possible InDels; then the Indel Realigner command was used to re-compare all the InDel reads in the neighborhood to improve the accuracy of SNP prediction. To ensure the reliability of SNPs, the obtained SNPs were further filtered with the following criteria: (1) Fisher’s test of strand bias (FS) ≤ 60; (2) haplotype score ≤ 13.0; (3) mapping quality (MQ) ≥ 40; (4) quality depth (QD) ≥ 2; (5) ReadPosRankSum ≥ −8.0; (6) MQ rank sum > −12.5 [67]. The detected SNPs were counted and the SNP loci were annotated using ANNOVAR software (Version v2016Feb01).

### 4.4. Population Genetic Diversity Analysis

The genetic diversity of the three tilapia populations was analyzed using the ‘populations’ command in the Stacks package (v.1.46). The following genetic diversity indices were calculated for the three populations: mean observed heterozygosity and homozygosity; mean expected heterozygosity and homozygosity; mean nucleotide polymorphisms (*Pi*); and mean population inbreeding factor (*F_IS_*). The fixation index (*F_ST_*) was also calculated for the three populations.

### 4.5. Population Genetic Structure and Population Kinship Analysis

The phylogenetic tree was constructed using the fastTree tool (http://www.microbesonline.org/fasttree/, accessed on 6 October 2024). This analysis was based on the SNP statistics of individuals from the three populations and measured the proximity of kinship. Genome-wide Complex Trait Analysis (GCTA) software (https://yanglab.westlake.edu.cn/software/gcta/#Overview, accessed on 6 October 2024) was used to remove SNP loci with minor allele frequency (MAF) <5%. A principal component analysis (PCA) was performed using data from individuals from the three populations. The genetic structure of the population was analyzed using Admixture software (http://dalexander.github.io/admixture, accessed on 6 October 2024), with the number of subpopulations set from two to ten (*K* = 2−10). The cross-validation error (CV error) was calculated for each *K* value to determine the optimal number of subpopulations. The group kinship was analyzed using the genome-wide relationship matrix (Gmatrix Ver.2) and identity-by-state (IBS) matrix (Plink v1.9) analyses.

### 4.6. Gene Flow and Linkage Disequilibrium Analyses

Next, among the three tilapia populations, we analyzed the gene flow that had potentially altered the populations’ gene pools. TreeMix (v 1.13) software was used to infer population splitting and mixing events based on the frequency of allele occurrence at SNP loci in the three populations, to visualize the populations’ maximum likelihood tree, and to identify the direction of gene drift in the populations. A linkage disequilibrium (LD) analysis was conducted on three tilapia populations to calculate the frequency of non-random association between alleles at different loci within the population. The LD metrics between different loci were expressed by the D value. Because the D value is greatly affected by allele frequency, normalized D’ and r^2^ values were used to measure the LD of the populations. PopLDdecay software (https://github.com/BGI-shenzhen/PopLDdecay, accessed on 6 October 2024) was used to calculate r^2^ values and to plot population LD decay.

### 4.7. Identification of Selected Genomic Regions and Gene Enrichment Analysis

The genomic regions that have been subject to significant selection were identified on the basis of nucleotide diversity (θπ) and *F_ST_* values obtained in pairwise comparisons of the three populations (OA, ON, and OS). The top 5% of SNP sites identified on the basis of θπ ratios (πOA/πON; πOA/πOS; πON/πOS) and *F_ST_* values were selected as significantly selected regions. Both the θπ values and *F_ST_* values were based on pre-filtered SNPs in 1 Mb non-overlapping windows on the genome in 100 kb sliding steps.

The functional enrichment analysis of genes in the selected regions was performed. Gene Ontology (GO) enrichment analysis was conducted using the R package TopGO version 2.36.0, and the *p*-value was calculated by the hypergeometric distribution method. The GO terms with a significance level of *p* < 0.05 were considered to be significantly enriched compared with the whole genome background. The Kyoto Encyclopedia of Genes and Genomes (KEGG) pathways associated with genes in the selected regions were identified by sorting the enrichment status according to Rich factor values, which was accomplished using the KEGG Automatic Annotation Server (KAAS) system.

### 4.8. Core SNP Loci Screening and Fingerprint Construction

According to genomic differences at the SNP loci detected in the three tilapia populations, the core SNP loci were further screened to construct SNP fingerprints for population identification. Referring to the filtering criteria of Wang et al. [68], core SNP sites were screened using Plink (v1.90b6.24). First, Indel loci were removed, leaving only the SNP loci on 22 chromosomes. The loci with minor allele frequency (MAF) > 0.2, heterozygosity (het) < 0.05, and deletion rate (geno) < 0.1 were retained. The SNP loci in intergenic regions (as determined from SNP annotation information) were removed. After genotype filling using Beagle (v.5.1) software, loci with MAF > 0.3 and het < 0.01 were retained; finally, the loci with LD r^2^ < 0.2 were screened. After obtaining the core SNP loci, the fingerprints corresponding to core SNP loci were plotted using the matplotlib graphic library of Python3.

### 4.9. SNP Fingerprint Validation and Determination of SNP Marker Combinations for Population Identification

To initially differentiate the three species of tilapia, the screened SNP loci were compared with the SNP fingerprints of the three species. To verify the accuracy of SNP fingerprinting, another 40 samples of caudal fins of blue tilapia, Nile tilapia, and red tilapia were collected, and genomic DNA was extracted as described in Section 2.2. The flanking sequences within 400 bp of the core SNP locus were extracted, and Primer 3.0 software (https://primer3.ut.ee/, accessed on 2 December 2024) was used to design specific amplification primers for each core SNP locus (Table S1). The DNA samples were used as templates for polymerase chain reaction (PCR) amplification reactions. The PCR amplification system and reaction procedures are listed in Table S2. The amplified products were sent to Tianlin Biological Co., Ltd. (Wuxi, China) for Sanger sequencing, and the sequencing results were used to accurately identify the genotypes of the SNP loci using Chromas Setup (v2.6.5) software, and to derive the corresponding amplified sequences. After verifying the fingerprinting patterns of the core SNP loci, the SNP marker combinations that could accurately identify blue tilapia, Nile tilapia, and red tilapia were selected from the core loci.

## 5. Conclusions

In this study, genetic differences among three major tilapia selection populations in China were detected with high precision. The red tilapia population had the highest genetic diversity and the blue tilapia population had the lowest genetic diversity. The three populations showed a significant tendency of genetic segregation, but the Nile tilapia population was more closely related to the red tilapia population. The three populations were mined for traits related to energy metabolism, growth and development, disease resistance, and reproduction that have been subject to selection during the process of adaptation to the natural environment and artificial selection. Meanwhile, SNP fingerprints for the accurate identification of wild Nile tilapia with the selected populations of blue tilapia and red tilapia were constructed based on the five core SNP markers, and two combinations of SNP markers for stock identification were developed for the first time. These findings highlight the genetic differences among these tilapia populations and will be useful for genetic improvement in breeding programs and for germplasm identification.

## Data Availability

All data generated or analyzed during this study are included in this published article and its Supplementary Materials.

## Supplementary Materials

The following supporting information can be downloaded at: https://www.mdpi.com/article/10.3390/ijms26104910/s1.
