# Supplementary material for: Genetic Structure, Selective Signatures, and Single Nucleotide Polymorphism Fingerprints of Blue Tilapia (Oreochromis aureus), Nile Tilapia Oreochromis niloticus), and Red Tilapia (Oreochromis spp.), as Determined by Whole-Genome Resequencing
Source: Int J Mol Sci. 2025 May 20;26(10):4910. doi: 10.3390/ijms26104910 (PMC12112444; doi:10.3390/ijms26104910)
Supplement: Supplementary file 1 [file ijms-26-04910-s001.zip › ijms-3562717-supplementary/Supplemental files/Supplemental Figure.pdf]

Supplemental Figures

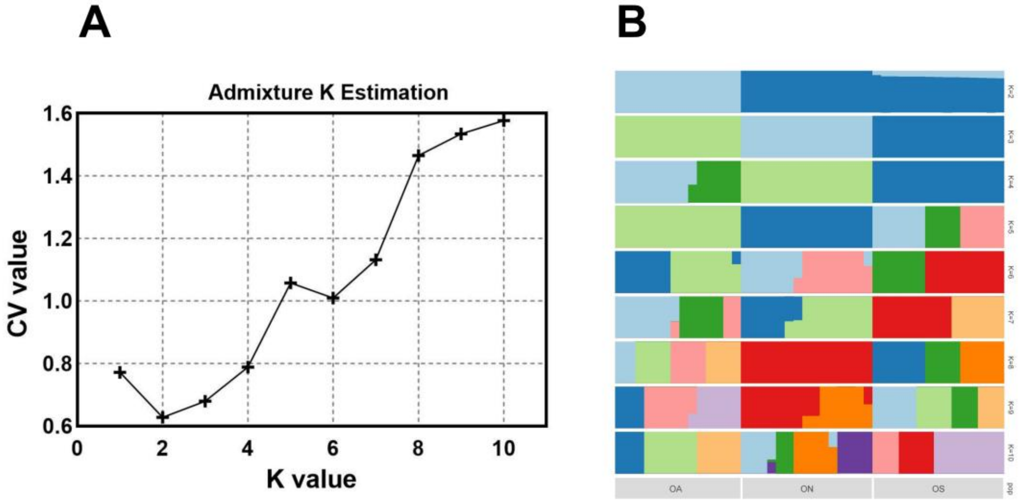

Fig. S1. (A) Distribution of CV error values corresponding to different K values; (B) Population genetic structure represented by different K values for the three populations

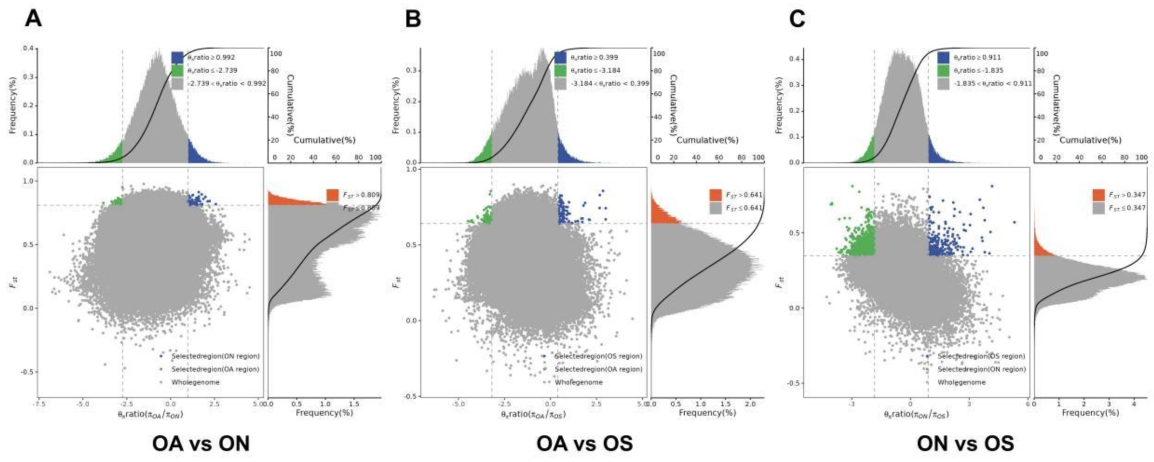

Fig. S2. Selection signal analysis of three groups (OA/ON, OA/OS, and ON/OS).
