# Supplementary material for: Genetic Structure, Selective Signatures, and Single Nucleotide Polymorphism Fingerprints of Blue Tilapia (Oreochromis aureus), Nile Tilapia Oreochromis niloticus), and Red Tilapia (Oreochromis spp.), as Determined by Whole-Genome Resequencing
Source: Int J Mol Sci. 2025 May 20;26(10):4910. doi: 10.3390/ijms26104910 (PMC12112444; doi:10.3390/ijms26104910)
Supplement: Supplementary file 1 [file ijms-26-04910-s001.zip › ijms-3562717-supplementary/Supplemental files/Supplementary Tables.pdf]

Supplemental Tables

Table S1. SNP marker-specific amplification primers for Sanger sequencing

SNP locus	Primer sequence (5′-3′)	TM (°C)	Product size (bp)
SNP1	F:AGGGATTCAAACACGGACAG<br>R:CTGGAGAGGAGGATACGCAG	59.97/60.0	323
SNP2	F:GCTGAACAGATAAGCTGGGC<br>AGCTGTACCTTTGGGGTCCT	59.99/60.0	242
SNP3	F:GGCAAATGAAGAGGGATGAA<br>R:aaatgtgtggagcacaccaa	60.0/60.0	469
SNP4	F:GGTCAACACATCAGTCCACG<br>R:GCACTGAAGACGGAGTCACA	60.0/60.0	760
SNP5	F:TGGAGGAACTCCTCGATGTC<br>R:CCTTGTTAACAGAGAGCGGC	60.2/60.0	323
SNP6	F:TTGCTCCTTCTTTGTGCCTT<br>R:TGAGGGTGTTGCCATAGACA	60.0/60.1	253
SNP7	F:AGGCCGATGACATTGCTAAC<br>R:TTGACAGGCAGTCACAGGAG	60.1/60.0	390

Table S2. (a) PCR reaction system. (b) PCR reaction program.

(a)

Total	ddH_2O	Primers	Premix Ex Taq	DNA
20μl	8μl	1μl	10μl	1μl

(b)

	Temperature	Time	Cycles
Pre-denaturation	95℃	5min	1 cycle
Denaturation	95℃	30s	
Annealing	52℃	30s	35 cycles
Extension	72℃	50s	
Extension	72℃	5min	

Table S3. Statistics of whole genome resequencing results of three populations.

Sample	HQReads	Reads	GC(%)	Q20(%)	Q30(%)	Average sequencingdepth
OA1	7921566	8123336(97.52)	41.82	98.85	96.95	4.18
OA2	8040308	8251184(97.44)	42.02	98.85	96.93	4.20
OA3	7925620	8124970(97.55)	41.80	98.85	96.95	4.18
OA4	6818814	7012598(97.24)	42.27	98.81	96.86	4.02
OA5	7066656	7253488(97.42)	41.82	98.83	96.86	4.05
OA6	7553384	7748456(97.48)	41.82	98.85	96.96	4.13
OA7	8062516	8270742(97.48)	41.91	98.83	96.90	4.20
OA8	5918210	6070508(97.49)	41.84	98.85	96.94	3.88
OA9	6713514	6890744(97.43)	42.00	98.81	96.86	4.00
OA10	6022862	6176290(97.52)	42.12	98.84	96.98	3.90
OA11	6932952	7110530(97.50)	41.86	98.84	96.95	4.03
OA12	6493532	6658332(97.52)	41.92	98.86	96.98	3.97
OA13	6211638	6376036(97.42)	42.06	98.81	96.91	3.92
OA14	8322870	8553104(97.31)	41.70	98.71	96.56	4.24
OA15	6571422	6720198(97.79)	41.68	98.88	97.02	3.98
Total	106575864	109340516(97.47)				
Average	7105058	7289368(97.47)	41.91	98.83	96.91	4.06
ON1	7927954	8406836(94.30)	42.56	97.14	94.87	4.18
ON2	8863966	9385182(94.45)	41.57	97.45	95.31	4.32
ON3	10574250	11240690(94.07)	49.91	97.11	94.85	4.57
ON4	8651762	9181800(94.23)	42.25	97.14	94.88	4.28
ON5	8497834	9024686(94.16)	41.87	97.08	94.78	4.26
ON6	9080092	9645952(94.13)	41.93	97.12	94.86	4.35
ON7	9275744	9851090(94.16)	41.97	97.13	94.88	4.38
ON8	9032766	9569992(94.39)	45.13	97.16	94.94	4.34
ON9	9585792	10202686(93.95)	41.95	97.00	94.64	4.42
ON10	8703882	9274220(93.85)	42.08	96.88	94.41	4.29
ON11	9640588	10309768(93.51)	43.89	96.93	94.54	4.43
ON12	8879474	9430912(94.15)	43.45	97.16	94.97	4.31
ON13	7995662	8497152(94.10)	41.93	97.11	94.84	4.19
ON14	10717994	11299024(94.86)	43.85	97.08	94.69	4.59

ON15	8400770	8874654(94.66)	41.85	97.24	95.07	4.25
Total	135828530	144194644(94.20)				
Average	90552358	9612976(94.20)	43.08	97.12	94.84	4.34
OS1	6665358	6817852(97.76)	41.65	98.94	97.13	3.99
OS2	7590008	7762014(97.78)	41.64	98.91	97.05	4.13
OS3	7592070	7776048(97.63)	41.76	98.90	97.05	4.13
OS4	8583880	8763500(97.95)	41.53	98.96	97.19	4.28
OS5	7432804	7603616(97.75)	41.76	98.93	97.11	4.11
OS6	7034622	7196264(97.75)	41.69	98.90	97.05	4.05
OS7	7023956	7197028(97.6)	41.74	98.88	96.97	4.05
OS8	7846482	8046510(97.51)	42.22	98.87	96.96	4.17
OS9	8156894	8351116(97.67)	41.89	98.92	97.10	4.22
OS10	8241802	8445832(97.58)	41.80	98.88	96.98	4.23
OS11	7852350	8041138(97.65)	41.74	98.90	97.03	4.17
OS12	8061128	8285038(97.30)	42.03	98.81	96.82	4.20
OS13	6580976	6744520(97.58)	41.81	98.89	97.04	3.98
OS14	10133978	10387172(97.56)	41.65	98.85	96.92	4.51
OS15	7897728	8102998(97.47)	41.77	98.85	96.92	4.18
Total	116694036	119520646(97.64)				
Average	7779602	7968043(97.64)	41.78	98.89	97.02	4.16

Table S4. Statistics of whole genome resequencing SNP results for three populations.

Sample	Holozygous	Heterozygous	Ts	Tv	Ts/Tv
OA1	4457230	307599	1970196	1109739	1.7754
OA2	4447907	289034	2000396	1124201	1.7794
OA3	4389733	277999	1979844	1117838	1.7711
OA4	4042974	250084	1764267	987588	1.7864
OA5	4205271	258938	1867170	1052149	1.7746
OA6	4257552	245242	1903364	1073057	1.7738
OA7	4459398	268833	1996210	1126426	1.7722
OA8	3722901	176560	1604474	900903	1.781
OA9	4074592	243105	1779036	998783	1.7812
OA10	3749230	183968	1630180	914203	1.7832
OA11	4123149	233456	1813798	1021604	1.7754
OA12	3998233	217530	1739415	978214	1.7782
OA13	3840431	205068	1674676	941026	1.7796
OA14	4690564	349806	2100807	1188501	1.7676
OA15	3995248	214536	1736100	977355	1.7763
Average	4163628	248117	1837329	1034106	1.7770
Total	62454413	3721758	27559933	15511587	
Sample	Holozygous	Heterozygous	Ts	Tv	Ts/Tv
ON1	6396259	333302	1078100	624172	1.7272
ON2	7153456	428095	1231932	711140	1.7323
ON3	9770355	1380577	2118938	1260862	1.6805
ON4	6981958	421527	1207618	693319	1.7418
ON5	7010402	425653	1215639	699618	1.7376
ON6	7198092	464392	1247492	718956	1.7351
ON7	7268647	465378	1271424	732738	1.7352
ON8	9922980	1620212	2255597	1344918	1.6771
ON9	7431334	469137	1281097	739153	1.7332
ON10	9796088	1614977	2243388	1335717	1.6795
ON11	6815550	392767	1174833	677927	1.733
ON12	6111375	312666	1031629	598525	1.7236
ON13	6815531	381025	1156950	662406	1.7466

ON14	6975738	437784	1214693	694412	1.7492
ON15	6938480	406062	1188698	683784	1.7384
Average	7505750	636904	1394535	811843	1.7247
Total	112586245	9553554	20918028	12177647	
Sample					
OS1	5294881	369487	1117578	633763	1.7634
OS2	5650732	466480	1256043	716972	1.7519
OS3	5738212	454069	1199722	685844	1.7493
OS4	5997057	541962	1382698	792149	1.7455
OS5	5788496	394861	1162770	659765	1.7624
OS6	5339044	402726	1177797	670491	1.7566
OS7	5349596	428894	1222834	692595	1.7656
OS8	5744259	525573	1342404	758392	1.7701
OS9	5855290	491109	1284311	731295	1.7562
OS10	5955046	492121	1275487	725583	1.7579
OS11	5722591	496514	1318343	750100	1.7576
OS12	5894337	517412	1314029	746172	1.761
OS13	5235669	390492	1140282	648622	1.758
OS14	6632976	759059	1622349	925323	1.7533
OS15	5887070	496045	1271799	725016	1.7542
Average	5739017	481787	1272563	724139	1.7575
Total	86085256	7226804	19088446	10862082	

Table S5. Seven SNP markers for chromosome location and mutation information.

SNP locus	Chr	Pos	Allele A	Allele B
SNP1	NC_031973.2	25098070	C	T
SNP2	NC_031978.2	17644090	G	A
SNP3	NC_031978.2	27379890	C	T
SNP4	NC_031979.2	40005392	G	A
SNP5	NC_031980.2	10766350	C	G
SNP6	NC_031981.2	23219247	G	A
SNP7	NC_031967.2	29863952	T	C
